# Pegylated Gold Nanoparticles Conjugated with siRNA: Complexes Formation and Cytotoxicity

**DOI:** 10.3390/ijms24076638

**Published:** 2023-04-02

**Authors:** Elżbieta Okła, Piotr Białecki, Marta Kędzierska, Elżbieta Pędziwiatr-Werbicka, Katarzyna Miłowska, Samuel Takvor, Rafael Gómez, Francisco Javier de la Mata, Maria Bryszewska, Maksim Ionov

**Affiliations:** 1 Department of General Biophysics, Faculty of Biology and Environmental Protection, University of Lodz, 141/143 Pomorska St., 90-236 Lodz, Poland; 2 Department of Organic and Inorganic Chemistry, Research Chemistry Institute “Andrés M. del Río” (IQAR), University of Alcalá, 28871 Alcalá de Henares, Spain; 3 Networking Research Center for Bioengineering, Biomaterials and Nanomedicine (CIBER-BBN), 28029 Madrid, Spain; 4 Institute “Ramón y Cajal” for Health Research (IRYCIS), 28034 Madrid, Spain

**Keywords:** gold nanoparticles, siRNA, complex formation, biophysical interaction, cytotoxicity

## Abstract

Drug delivery systems such as dendrimers, liposomes, polymers or gold/silver nanoparticles could be used to advance modern medicine. One significant pharmacological problem is crossing biological barriers by commonly used drugs, e.g., in the treatment of neurodegenerative diseases, which have a problem of the blood-brain barrier (BBB) restricting drug delivery. Numerous studies have been conducted to find appropriate drug carriers that are safe, biocompatible and efficient. In this work, we evaluate pegylated gold nanoparticles AuNP14a and AuNP14b after their conjugation with therapeutic siRNA directed against APOE4. This genetic risk factor remains the strongest predictor for late-onset Alzheimer’s disease. The study aimed to assess the biophysical properties of AuNPs/siAPOE complexes and to check their biological safety on healthy cells using human brain endothelial cells (HBEC-5i). Techniques such as fluorescence polarization, circular dichroism, dynamic light scattering, ζ-potential measurements and gel retardation assay showed that AuNPs form stable complexes with siRNA. Subsequently, cytotoxicity assays proved the biological safety of formed conjugates. Obtained results enabled us to find effective concentrations of AuNPs when complexes are formed and non-toxic for healthy cells. One of the studied nanoparticles, AuNP14b complexed with siRNA, displayed lower cytotoxicity (MTT assay, cells viability −74.8 ± 3.1%) than free nanoparticles (44.7 ± 3.6%). This may be promising for further investigations in nucleic acid delivery and could have practical use in treating neurodegenerative diseases.

## 1. Introduction

Nanotechnology is a branch of science that finds many applications in many fields of study, such as biomedicine, engineering, chemistry, and physics. For example, nanoparticles can be used as antibacterial agents, biosensors, pollution removals, and immunostimulatoros in vaccines and drug delivery systems [1,2]. Furthermore, modern biomedical nanosystems can deliver drugs by passive or self-delivery. The first relies on the hydrophobic effect and drug encapsulation into nanoparticle cavities, and the second consists of the direct conjugation of the targeted molecule [3,4]. Also, the targeting of delivered structures can be divided into active and passive. Active targeting has a more elaborate mechanism of action, where administrated complex binds to specific receptors in the site of action. On the other hand, complex affinity to the binding site or its biophysical properties is used in passive targeting, where nanostructure with drug adrift in the bloodstream [3].

Gold Nanoparticles (AuNPs) are promising carriers for different biomolecules such as drugs, therapeutic proteins, DNA or RNA. AuNPs’ size-to-volume ratio, positive charge and ability to protect the payload molecule from degradation or undesired immunological reaction could play a key role in biomedicine [5]. They can also be metabolized in lysosomes, where they dissolute ROS activity [6]. Modifications such as the formation of silica shells, protein attachments and pegylation have been studied to make these nanoparticles stable, safe and efficient [7]. AuNPs covered with PEG (polyethylene glycol) are more durable because PEG prevents them from aggregation.

Moreover, the presence of PEG increases solubility in water and cellular uptake and avoids adverse immunological responses. However, the outer layer of PEG can impact the AuNP positive charge leading to the obstruction of complexation [8,9]. Therefore, it is essential to establish the best protocol and crucial PEG concentration [10,11,12]. Furthermore, adding PEG into the gold nanoparticle structure can facilitate the passage of nanoparticles through different biological barriers such as mucus (e.g., in the airways or vagina) or the extracellular matrix in the brain tissue after crossing the blood-brain barrier [13,14,15]. PEG-nanoparticles’ ability to impact the brain tissue makes them worthy of study in brain delivery systems.

This study focused on pegylated gold nanoparticles as the potential carriers for therapeutical small interfering RNA (siRNA) directed towards the apolipoprotein E gene (ApoE). One of the alleles of this gene, ApoE4, is related to the onset of Alzheimer’s disease. This mechanism is connected to an excessive β-amyloid deposition with simultaneous poor clearance of these peptides [16]. However, siRNA alone is very unstable, has unsatisfactory pharmacokinetic properties and can have non-intentional effects [17]. To make it more stable, siRNA was submitted to some chemical modifications where the siRNA complexation with cationic nanoparticles, such as AuNPs, presents a wide range of benefits. The siRNA sealed by the nanoparticle was more likely to arrive at its target and not be destroyed by the external environment or detected by the immunological system. Moreover, the outer positive charge facilitated the cellular uptake of the complex [18]. AuNPs can also support the siRNA escape from endosomes [19,20].

To assess AuNP/siRNA-*APOE4* complexation, we chose 2 gold nanoparticles, AuNP14a and AuNP14b, characterized in previous publications [21,22,23]. Both of these compounds are cationic dendronized gold nanoparticles coated with PEG. However, they differ in the dendron/PEG ratio, which for AuNP14a is higher (3:1) than for AuNP14b (1:1). Because a different amount of PEG on the particle surface may change its characteristics and mode of action, [24] it was decided to check which nanoparticle would have better effects as a potential carrier. The aim of this study is the examination of the biophysical characterization of the AuNP/siRNA complexes and whether they are safe and efficient to further investigation for the application in the treatment of neurodegenerative diseases. The preliminary data displayed below focuses on determining the AuNP/siRNA complexation ratio, nanoparticle impact on siRNA structure, and cytotoxicity assessment of the tested complexes. Low cytotoxicity and affinity to nucleic acid suggest that tested nanoparticles can be investigated as potential carriers of therapeutical siRNA. This research is the first that presents these specific AuNPs conjugated with siAPOE4, playing an essential role in the onset of neurodegenerative diseases, proving that these kinds of complexes could be safe for healthy cells.

## 2. Results and Discussion

### 2.1. Zeta Potential and Hydrodynamic Diameter

**Figure 1:**
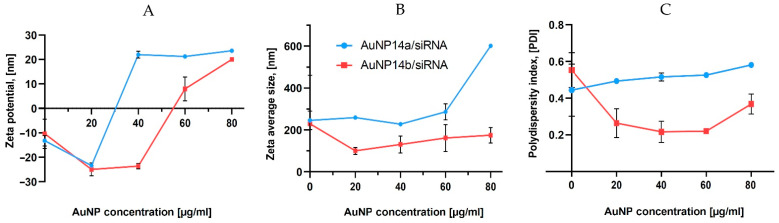
Zeta potential (**A**), hydrodynamic diameter (**B**) and polydispersity index (**C**) of siRNA alone and in the presence of increasing amounts of studied gold nanoparticles. Measurements were performed by the titration method. siRN concentration, 1 µM. The results represent mean values with standard deviation (*n* = 3).

The zeta potential of AuNP/siRNA complexes was measured in a constant siRNA concentration (1 µmol/L) with different nanoparticle concentrations ranging from 20 to 80 µg/mL (Figure 1A). This study assessed the AuNP concentration needed for the complex saturation. ζ-potential curves crossed “0” toward positive values when the nanoparticle concentrations reached 30 µg/mL (AuNP14a) and 55 µg/mL (AuNP14b). This difference can be explained by the higher positive charge of nanoparticle AuNP14a. The positive charge of a compound facilitates cell uptake but also increases nanoparticle cytotoxicity [25]. Therefore, finding the optimal concentration for efficient complexation with siRNA and safe application is essential.

Dynamic light scattering (DLS) measurements were performed to assess the AuNPs/siRNA hydrodynamic diameter in relation to increasing nanoparticle concentration. In the case of AuNP14a, the hydrodynamic diameter was around 250 nm, while for AuNP14b it was 150 nm (Figure 1B). DLS data for free siRNA shows relatively large sizes, in line with other reports [25,26,27], and can be explained by the formation of higher-sized aggregates by noncomplexed siRNA [28]. Accordingly, the PDI of measured complexes increased with higher amounts of AuNPs (Figure 1C), showing that bigger complexes become less monodispersed and have a higher tendency for aggregation [29].

### 2.2. Fluorescence Polarization

**Figure 2:**
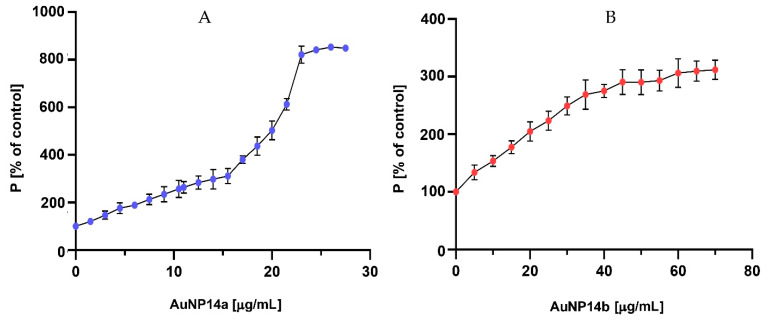
Changes in siRNA-FL fluorescence polarization after titration with (**A**) AuNP14a and (**B**) AuNP14b. Results of samples containing AuNPs presented as [%] of siRNA values without nanoparticles. siRNA concentration 1 μmol/L in sodium phosphate buffer 10 mmol/L, pH 7.4. λexc = 485 nm, λem = 516 nm. Results are mean ± standard deviation (SD), *n* = 3.

The fluorescence polarization technique was used to confirm the ability of the tested gold nanoparticles to complex with siRNA. When a complex is formed, the fluorescently labelled siRNA movement in solvent slows, fluorescence intensity drops, and polarization increases [30]. Polarization results indicate that the complexation ratios are compatible with those established by zeta potential measurements. The measurement benchmark was a polarization obtained for 1 µmol/L of siRNA-FL in phosphate buffer, presented as 100%. The plateau phase was reached at 23–25 µg/mL concentration for AuNP14b (Figure 2A) and 54–60 µg/mL for AuNP14b (Figure 2B). Results confirm the hypothesis that a greater amount of AuNP14b than AuNP14a is needed to form a complex. Comparing the results for both nanoparticles, it could be seen that AuNP14a, used in smaller concentrations, induced higher polarization values than AuNP14b. The molecular weight of ligands is one of the factors that can influence the degree of siRNA fluorescence polarization change [31]. AuNP14a, bigger with a higher dendron/PEG ratio, presented higher fluorescence polarization values in the plateau phase than the smaller AuNP14b.

### 2.3. Agarose Gel Electrophoresis

**Figure 3:**
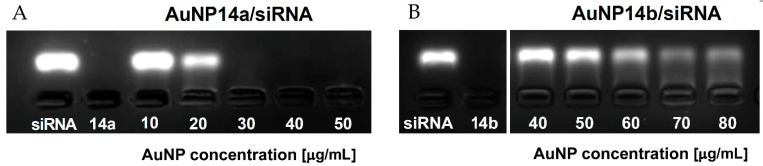
3% Agarose gel electropherograms of ApoE4 siRNA complexed with gold nanoparticles, (**A**) AuNP14a and (**B**) AuNP14b. Samples containing 1 µmol/L siRNA per line and nanoparticles applied in the corresponding concentrations were prepared in sodium phosphate buffer 10 mmol/L in the presence of GelRed stain. Gels were visualized upon transillumination at 525 nm.

Gel electrophoresis is another simple but precise method, which helps to determine the character of interaction between siRNA and AuNPs and establish the appropriate concentrations of ligand needed to form a fully saturated complex with siRNA. Fluorescence decay can indicate that nonlabelled nanoparticles bind to siRNA molecules and slow down the movement of the negatively charged siRNA through the gel. When concentrations of the nanoparticle are high enough to saturate the complex, siRNA cannot migrate towards the anode due to the loss of negative charge by the complexes and the increased size [32]. In the case of AuNP14a, it was observed that the fluorescence band disappeared between the concentrations of 20 and 30 µg/mL, with this result matching other applied methods (Figure 3A). The electrophoregram of AuNP14b (Figure 3B) shows that the nanoparticle could not saturate the complex in the same concentrations and quenched the fluorescence signal in concentrations above 60 µg/mL. This effect might have been caused by the smaller size and lower molecular weight of AuNP14b, similar to the impact of other nanoparticles reported in [26,33]. As the concentration of AuNP14b increases, the siRNA band starts to decline.

Gel retardation assay was also applied to analyze the time stability of studied complexes. AuNP14a/siRNA complexes were prepared 48 h, 24 h and 1 h before electrophoresis in the conditions described above. Results show that studied nanoconjugates are stable in time (Figure S1).

### 2.4. Circular Dichroism

**Figure 4:**
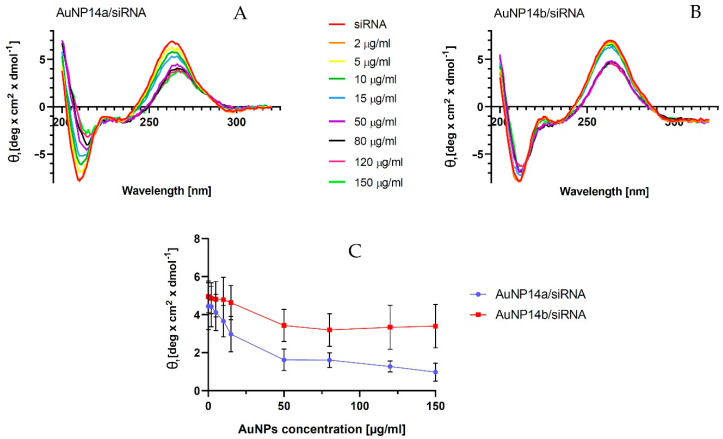
Effect of gold nanoparticles on siRNA’s secondary structure (θ—ellipticity). (**A**) Spectra CD of siRNA at the presence of AuNP14a, and (**B**) AuNP14b. (**C**) Changes in mean ellipticity at λ = 265 nm. Measurements were conducted in λ = 200–320 nm in phosphate buffer, pH = 7.4. The concentration of siRNA 1 µmol/L. The values are the mean ± standard error of the mean (SEM), *n* = 3.

Measurements of circular dichroism (CD) were performed to investigate the changes in the secondary structure of siRNA. The complexation of siRNA with the nanoparticle changes with the siRNA secondary structure. A CD technique enables an investigation of the binding of tested molecules to siRNA without using any probes [34,35]. The titration of nanoparticles led to decreases in siRNA ellipticity yield. This effect showed the complexation between AuNP and siRNA molecules [36]. In the case of tested gold nanoparticles, we observed that the ellipticity decreases specifically for siRNA peaks at λ = 265 nm and increases at λ = 210 nm (Figure 4A,B). The spectra shift to the right visible on graphs may represent slight structural changes in the siRNA secondary structure. Obtained results showed that AuNP14a generated a more visible shift and possibly caused bigger structural changes in nucleic acid than AuNP14b. In addition, AuNP14b in higher concentrations seemed to form more stable complexes with the siRNA than AuNP14a, maintaining a constant level of ellipticity and spectra shifting. The CD results, expressed as molar ellipticity Ɵ (deg × cm^2^ × dmol^−1^), confirmed that both gold nanoparticles interacted with siRNA due to the changes in the ellipticity. However, the interaction had a small impact on the secondary structure of nucleic acid and did not modify its biological activity, as confirmed by the data presented in Figure 4C. representing the changes in mean ellipticity at λ = 265 nm.

### 2.5. Cytotoxicity

**Figure 5:**
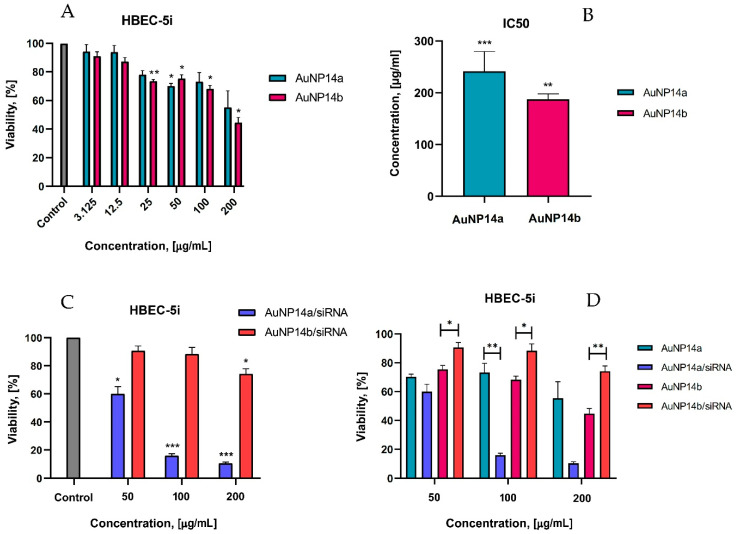
Cytotoxicity effect of AuNPs alone and their complexes with siRNA toward HBEC-5i cells, MTT assay. (**A**) The viability of HBEC-5i cells in the presence of AuNPs at the concentrations varied from 1.56 to 200 µg/mL. Statistical significance showed when compared with the control, non-treated cells. (**B**) Calculated from the results shown on panel A, IC_50_, values of noncomplexed nanoparticles vs. control (100% viability). (**C**) The viability of HBEC-5i cells in the presence of AuNPs/siRNA complexes. Significant differences compared to the negative control. (**D**) Comparison of the cytotoxic effects of naked AuNPs and their complexes with siRNA. Statistical significance was marked for the same concentrations between AuNPs alone and in the complex with siRNA. To form the AuNP/siRNA complex, the concentration of siRNA was always 1 µmol/L, whereas the concentration of nanoparticles was varied—incubation time for all samples 24 h. Data points represent means ± SD obtained from min. 3 separate experiments. Statistical significance was assessed using a two-way ANOVA test (*n* = 3, * *p* < 0.05; ** *p* < 0.01, *** *p* < 0.0001).

**Figure 6:**
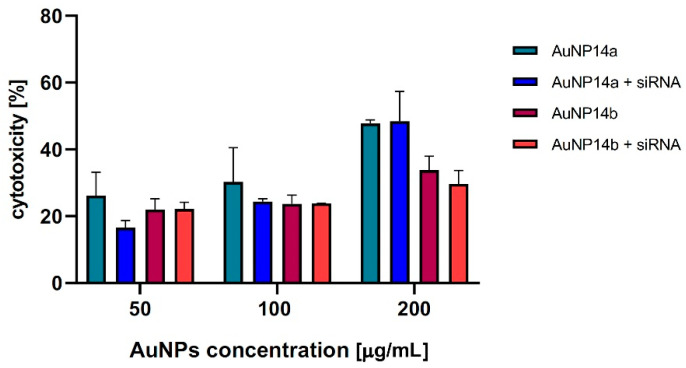
Cytotoxicity effect of AuNPs alone and complexes with siRNA toward HBEC-5i cells, LDH assay. The nanoparticles concentrations varied from 50 to 200 µg/mL. All results were compared to positive control with Triton X-100: incubation time, 24 h. Data points represent means ± SD obtained from min. 3 separate experiments.

Nanoparticles used as drug carriers and especially for gene delivery through the Blood Brain Barrier (BBB) must be non-toxic to human cells. Since AuNPs and their complexes with siRNA could be involved in treating brain diseases, human brain endothelial cells (HBEC-5i) were chosen to determine their toxicity. The HBEC-5i cell line was selected for this experiment because these cells can express the ApoE gene [37]. It was important to find the nontoxic or low toxic AuNP concentration for cells of the BBB and crucial in the case of tested AuNPs when considering their further testing in terms of potential delivery of siRNA to the brain. In addition, the viability of peripheral blood mononuclear cells (PBMC) in the presence of AuNP/siRNA complexes was assessed. PBMC cells can be used when the cytotoxicity of ligands is analyzed. In this study, MTT and LDH assays were performed for HBEC-5i cells (Figure 5). PBMC cell viability was tested with the Alamar Blue test (Figure 6) as with the previously described method [17]. This technique represents the ability of PBMC cells to metabolize resazurin after incubating with the tested compounds.

#### 2.5.1. Viability of HBEC-5i Cells by MTT Test

Our studies revealed that nanoparticles had no toxic effect on human brain endothelial cells at concentrations less than 12.5 µg/mL. According to studies, the cytotoxic effect is considered a ≥30% reduction of cells [38]. Only higher concentrations of nanoparticles (≥200 µg/mL) exhibited a strong cytotoxic effect (Figure 5A) showing a viability level of 55.4 ± 11.5% for AuNP14a and 44.7 ± 3.6% for AuNP14b. Compared with non-treated cells (negative controls), statistically significant effects were observed for AuNP14a in concentrations of 50 µg/mL and with a viability value of 70.3 ± 1.8%. For AuNP14b, all concentrations above 25 µg/mL (viability 73.6 ± 1.3%) were statistically significant and presented a cytotoxic effect at the highest concentration (44.7 ± 3.6%). For both nanoparticles, IC50 was defined (Figure 5B). The IC50 value for AuNP14a was higher than for AuNP14b, 241.5 ± 38 µg/mL and 187.3 ± 10.5 µg/mL, respectively. These proportions drastically changed, especially in the case of AuNP14a, when nanoparticles were complexed with siRNA (Figure 5C).

In the highest tested concentration of AuNP14a/siRNA, the complex was strongly destructive for cells, while AuNP14b conjugated with siRNA showed only mild cytotoxicity (10.4 ± 1% and 74.8 ± 3.1% respectively). The statistical analysis showed that complexation with siRNA decreases the toxicity of AuNP14b towards HBEC-5i but not for AuNP14a (Figure 5D).

#### 2.5.2. Viability of HBEC-5i Cells by LDH Assay

To confirm the results obtained by MTT assay, the additional viability tests were performed by LDH assay. The data presented in Figure 6 indicate that the presence of gold nanoparticles or AuNPs/siRNA complexes reduces the HBEC-5i viability. In addition, the results obtained in both tests showed a higher cytotoxicity of AuNP14a than AuNP14b, for which statistically significant changes in the case of the LDH test were determined for concentration 200 µg/mL. However, AuNP14b did not show any significant changes in cytotoxicity after complexation with siRNA.

#### 2.5.3. Viability of PBMCs Cells by Alamar Blue Assay

**Figure 7:**
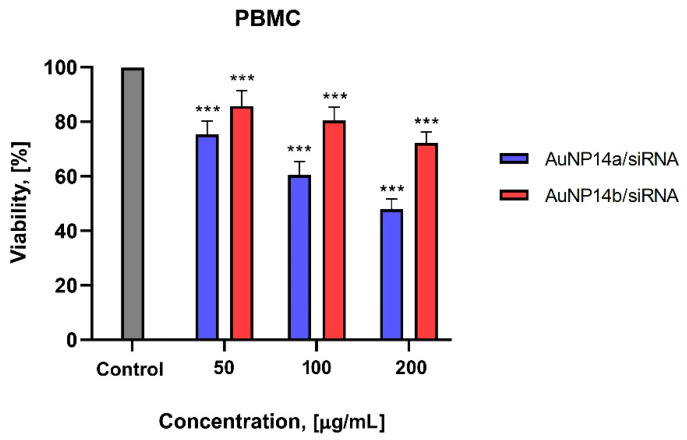
Viability of PBMC cells after incubating with AuNPs/siRNA complexes, Alamar Blue assay. Statistical effects showed in comparison to control cells. The concentration of siRNA is 1 µmol/L, incubation time 24 h. Data points represent means ± SD obtained from min. 3 separate experiments. Statistical significance was assessed using a two-way ANOVA test (*n* = 3, *** *p* < 0.0001).

The viability of PBMC cells tested using Alamar Blue assay after their incubation with AuNP/siRNA complexes showed lower cytotoxicity in comparison to previously described results. It was noticed that PBMC after treatment with 50 µg/mL of AuNP14a/siRNA showed a viability of 75.4 ± 5% and for AuNP14b/siRNA of 85.7 ± 5.7% (Figure 7).

Because AuNPs can interact with peripheral blood *mononuclear* cells (the major cells in the human body’s immunity) and change their response to the ligand action, it is crucial to maintain the high level of these cells after AuNPs administration [21,39].

Results obtained in this study confirm our hypothesis that studied gold nanoparticles could be considered in further investigations as a possible delivery platform for siRNA. Furthermore, the complexation and cytotoxicity tests indicate the optimum AuNP concentration that can be applied to form the AuNP/siRNA complexes is 50 µg/mL. These findings agree with previously published results that characterized gold nanoparticles as possible nanocarriers for nucleic acids [40,41,42].

## 3. Materials and Methods

### 3.1. Pegylated Gold Nanoparticles of the Second Generation

**Figure 8:**
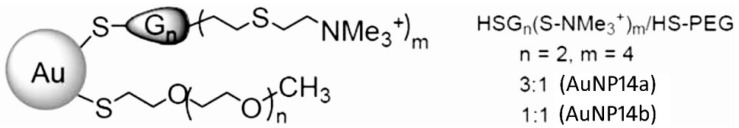
Structure of tested dendronized AuNPs modified with PEG. AuNPs were synthesized in water by the reaction of HAuCl_4_·3H_2_O with a mixture of two ligands containing a thiol moiety: (1) the cationic dendrons HSG_2_(SNMe_3_^+^)_4_ and (2) commercial PEG ligand CH_3_O(CH_2_CH_2_O)_n_CH_2_CH_2_SH, HS-PEG. In addition, NaBH_4_ was used as a reducing agent [21].

The biochemical characteristics and synthesis steps of gold nanoparticles containing polyethylene-glycol (PEG) and named AuNP14a and AuNP14b are described previously [21,22,23]. The AuNPs structure and characterization are present in Figure 8 and Table 1. The weight concentration (µg/mL) of AuNPs was used to study their biological effects, complexation characteristics and cytotoxic effects.

- AuNP14a, dendron/PEG molar ratio = 3:1
- AuNP14b, dendron/PEG molar ratio = 1:1

### 3.2. siRNA

Gold nanoparticles were complexed with non-fluorescent or FITC-labelled siRNA coding ApoE4 (Sense: 5′G.A.U.U.A.C.C.U.G.C.G.C.U.G.G.G.U.G.C.U.U. Antisense: 5′-P.G.C.A.C.C.C.A.G.C.G.C.A.G.G.U.A.A.U.C.U.U.). siRNA was purchased from Dharmacon Inc., Lafayette, CO, USA. siRNA was dissolved in 1xsiRNA buffer (Dharmacon Inc., Lafayette, CO, USA).

### 3.3. Materials

HBEC-5i cell line was purchased from ATCC (Manassas, VA, USA). DMEM-F12 and RPMI-1640 were acquired from Biowest, Nuaillé, France. Other reagents used in cell cultures (ECGS, 1% Penicillin-Streptomycin, FBS, MTT, Resazurin, Triton X-100) were purchased from Sigma-Aldrich, Saint Louis, MO, USA. DMSO used in the MTT assay was purchased from Avantor, Radnor, PA, USA. CyQUANT™ LDH Cytotoxicity Assay kit was acquired from InvitrogenTM Thermo Fisher Scientific, Waltham, MA, USA. Gelatin was obtained from Serva, Heidelberg, Germany. PBS used in experiments was prepared from tablets acquired from GibcoTM, Thermo Fisher Scientific, Waltham, MA, USA. While preparing gel electrophoresis, agarose from Maximus, Łódź, Poland and GelRed stain purchased from Biotium, Inc., Fremont, CA, USA, was used.

### 3.4. AuNp:siRNA Complex Formation

AuNps/siRNA complexes were formed using a previously reported protocol [43] with minor modifications. Appropriate volumes of siRNA and AuNPs dissolved in sodium phosphate buffer 10 mmol/L, pH 7.4 were mixed at the concentrations giving desired AuNP/siRNA molar ratios. The mixture was gently vortexed and incubated for 20 min at room temperature. Mixtures used for complex characterization or in vitro experiments were made in the same conditions (buffer, temperature, etc.). Complexes were prepared with autoclaved buffer and components. Solutions were kept in sterile conditions in the dark.

### 3.5. ζ-Potential and Hydrodynamic Diameter

Measurements of the potential and size of the particles were performed on a Malvern Zetasizer Nano ZS, Worcestershire (UK). Solutions of siRNA (1 µM) in PBS were prepared and titrated with AuNPs, up to 80 µg/mL. Zeta potential was estimated with Helmholtz–Smoluchowski’s equation. Results were collected from 5 measurements, 7 runs each and provided information about the complexation ratio. Any changes in the zeta potential of complexes in time (relative value estimated to incubation time, 15 min) were measured during 240 min to estimate the time stability of formed complexes. The zeta potential of AuNP/siRNA complexes was measured to assess the stability of the obtained complexes over time. The measurement was carried out for complexes formed by siRNA (constant concentration 1 µmol/L) and concentrations of nanoparticles, corresponding to the saturated complex C_AuNP14a_ = 60 ug/mL, C_AuNP14b_ = 130 ug/mL.

The size of gold nanoparticles was measured by the dynamic light scattering method. Backscatter was set for 173°, Abs = 720 nm, and refractive index (RI) was 0.28. The number of the performed measurements was 5, 3 runs each.

### 3.6. Fluorescence Polarization

As an alternate method to confirm the complexation ratio, 1 µM of siRNA-FL in a phosphate buffer (10 mmol/L, pH = 7.4) was prepared and measured in a PerkinElmer LS-50B spectrofluorometer (excitation: λ = 485 nm, emission: λ = 516 nm). siRNA was then titrated with increasing concentrations of the tested AuNPs.

### 3.7. Gel Retardation Assay

To check the appropriate concentrations of the components for the AuNP/siRNA complex formation, 3% agarose gel electrophoresis was performed. Complexes were prepared in PBS and incubated for 15 min at room temperature. The concentration of siRNA was constant (1 µmol/L). The negative control used a non-complexed nanoparticle in its highest applied concentration. Electrophoresis was run for 45 min (90 V, 35 mA) with GelRed stain (0.05 µg/1 mL). Results were analyzed using ChemiDoc-It^2^ Imager (UVP, Cambridge, UK).

### 3.8. Circular Dichroism (CD)

The circular dichroism technique was applied to analyze the changes in the siRNA secondary structure involved in the presence of tested gold nanoparticles. siRNA was titrated with the AuNPs in a concentration range 2–150 µg/mL. Changes in the ellipticity of the nucleic acid were checked using a Jasco J-815 CD spectrometer (Jasco International Co., Ltd., Tokyo, Japan). Baseline and probes were measured in phosphate buffer (10 mmol/L, pH = 7.4) at a wavelength set from 200 to 320 nm.

### 3.9. Cell Culture

In vitro experiments were conducted on human brain endothelial cells (HBEC-5i) and peripheral blood mononuclear cells (PBMC). HBEC-5i was purchased from ATCC and cultured in 10% FBS, 1% Penicillin-Streptomycin and DMEM-12F media enriched with 40 µg/mL endothelial cell growth supplement (ECGS). Culture flasks were covered with 1% gelatin. PBMCs were isolated from the buffy coat fraction from healthy blood donors obtained from Central Blood Bank and cultured in RPMI-1640 medium with 10% FBS with the addition of 1% antibiotic. Both cell lines were incubated under standard conditions (37 °C, 5% CO_2_).

### 3.10. Cytotoxicity

MTT test. The cytotoxicity of AuNPs and their complexes with siRNA towards the HBEC-5i cell line was tested using an MTT assay. Cells were seeded on 96-well plates in density 1 × 10^4^ cells/well and incubated under standard conditions. AuNPs or AuNP/siRNA complexes at 1–400 µg/mL concentration range were added to the cells and incubated for 24 h. MTT solution at the concentration 0.5 mg/mL in PBS was added for 3 h, 100 μL DMSO was added to the samples to dissolve the formazan crystals. Sample absorbance was measured at the wavelengths λ = 580 nm and λ = 720 nm using a multiwell plate reader (BioTek PowerWave HT, BioTek Instruments, Inc. Winooski, VT, USA). The cell viability percentage was calculated by the formula:

% viability = (A_s_/A_c_) × 100% (1)

where: A_s_—absorbance of treated cells, A_c_—absorbance of control cells.

Alamar blue assay. The cytotoxicity of AuNP complexes with siRNA was tested on PBMCs. Cells were seeded on 96-well black plates in density 1 × 10^4^ cells/well and incubated under standard conditions. Next, auNPs/siRNA in selected concentrations were added to the cells and incubated 24 h. After this time, resazurin solution in PBS was added to cells to obtain a final concentration of 0.125 mg/mL per well. After 2 h incubation, fluorescence was read at a multiwell plate reader with wavelengths set at λ_ex_ = 530 nm and λ_em_ = 590 nm. The cell viability percentage was calculated by the same formula as presented in the MTT assay.

LDH assay. The cytotoxicity of AuNPs and their complexes with siRNA towards the HBEC-5i cell line was additionally tested using an LDH assay. Cells were seeded on 96-well plates in a 1 × 10^4^ cells/well density and incubated under standard conditions. AuNPs or AuNP/siRNA complexes at 50–200 µg/mL concentrations were added to the cells and incubated for 24 h. The colorimetric test quantified lactate dehydrogenase, an enzyme released from damaged cells where lactate dehydrogenase reduces NAD+ to NADH, and NADH reduces tetrazole salts to a colored product—formazan. Absorbance measurement at λ = 490 nm was used to assess the amount of formazan formed. Cells were incubated for 24 h. After the time had elapsed, 10 μL of the test substance was added per well, and 50 μL of the medium was transferred to a new 96-well plate. A reaction mixture with CyQUANT LDH Cytotoxicity Assay Kit Substrate Mixture was prepared by dissolving it in 11.4 mL of distilled water, followed by the addition of 600 μL Assay Buffer Stock Solution. 50 μL of the reaction mixture was added to each well, and the plate was left for 30 min in the dark. Sample absorbance was measured at the wavelengths λ = 580 nm and λ = 720 nm using a multiwell plate reader (BioTek PowerWave HT, BioTek Instruments, Inc. Winooski, VT, USA). The cell viability percentage was calculated by the formula:

% viability = (As/Ac) × 100%(2)

where: A_s_—absorbance of treated cells, A_c_—absorbance of control cells

### 3.11. Statistical Analysis

The results are presented as mean values ± standard deviation (SD) of 3 independent experiments (n = 3). For CD results standard error of the mean (SEM) instead of SD was shown. The stability of complexes in time is presented as mean values ± standard deviation (SD) of 6 independent experiments (n = 6). To analyze the data Two-way Anova and a Mixed-effects analysis were applied. Statistical significance was taken as *p*-values that were considered statistically significant as follows: * *p <* 0.05, ** *p <* 0.01, *** *p <* 0.0001.

## 4. Conclusions

This work describes the biophysical properties and cytotoxicity profiles of 2 gold nanoparticles (AuNP14a and AuNP14b) conjugated with therapeutical siRNA directed toward the apolipoprotein E gene (ApoE). Both compounds were analysed and compared. The tested AuNPs were able to form complexes with anti-ApoE siRNA. Obtained results indicate that nanocomplexes formed by AuNP14b (1:1 dendron/PEG ratio) are more suitable as siRNA carriers when compared to the systems formed by AuNP14a (3:1 dendron/PEG ratio). The AuNP14b/siRNA system had an appropriate size, and PDI index desired for drug carriers and was less toxic towards human brain endothelial (HBEC-5i) and peripheral blood mononuclear cells (PBMC). The complexation of positive AuNPs with anionic nucleic acid positively impacted the nanoparticle toxicity against studied normal cells. Therefore, the compound AuNP14b should be further examined for its effective internalization, drug protection, release kinetic and reduced toxicity.

## Figures and Tables

**Table 1:** Characterization of gold nanoparticles considered in this work [21].

Nanoparticle	Solubility	Dendron/PEG Molar Ratio	^1^ ZP, (mV)	^2^ d, (nm)
AuNP14a	water	3/1	44.9	34.00
AuNP14b	water	1/1	41.1	23.3

^1^ Zeta potential; ^2^ zeta average size (DLS).

## Data Availability

Not applicable.

## Supplementary Materials

The following supporting information can be downloaded at: https://www.mdpi.com/article/10.3390/ijms24076638/s1.
