# Supplementary material for: Pegylated Gold Nanoparticles Conjugated with siRNA: Complexes Formation and Cytotoxicity
Source: Int J Mol Sci. 2023 Apr 2;24(7):6638. doi: 10.3390/ijms24076638 (PMC10094790; doi:10.3390/ijms24076638)
Supplement: Supplementary file 1 [file ijms-24-06638-s001.zip › ijms-2298481-supplementary.pdf]

Supporting Information

Article

Pegylated Gold Nanoparticles Conjugated with siRNA: Complexes Formation and Cytotoxicity

Elżbieta Okła [1], Piotr Białecki [1], Marta Kędzierska [1], Elżbieta Pędziwiatr-Werbicka [1], Katarzyna Miłowska [1], Samuel Takvor [2,3,4], Rafael Gómez [2,3,4], Francisco Javier de la Mata [2,3,4], Maria Bryszewska [1] and Maksim Ionov [1,*]

1 Department of General Biophysics, Faculty of Biology and Environmental Protection, University of Lodz, 141/143 Pomorska St., 90-236 Lodz, Poland
2 Department of Organic and Inorganic Chemistry, Research Chemistry Institute "Andrés M. del Río" (IQAR), University of Alcalá, 28871 Alcalá de Henares, Spain
3 Networking Research Center for Bioengineering, Biomaterials and Nanomedicine (CIBER-BBN), 28029 Madrid, Spain
4 Institute "Ramón y Cajal" for Health Research (IRYCIS), 28034 Madrid, Spain
* Correspondence: maksim.ionov@biol.uni.lodz.pl

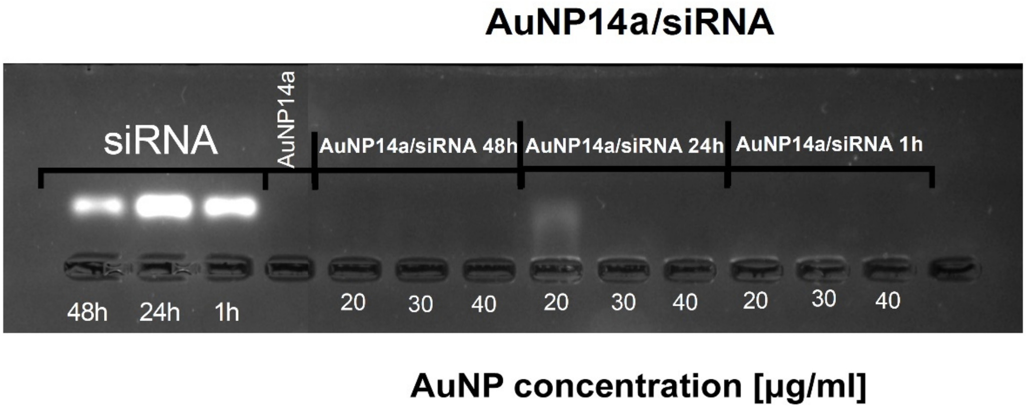

Figure S1. AuNP14a/siRNA complex stability in time (1 h, 24 h and 48 h). 3% Agarose gel electrophoregrams of ApoE4 siRNA alone or complexed with gold nanoparticle. Samples containing 1 µmol/L siRNA per line and nanoparticles applied in the corresponding concentrations were prepared in sodium phosphate buffer 10 mmol/L in the presence of GelRed stain. Gels were visualized upon transillumination at 525 nm.
